# The promoter and the enhancer region of the KLK 3 (prostate specific antigen) gene is frequently mutated in breast tumours and in breast carcinoma cell lines

**DOI:** 10.1038/sj.bjc.6690254

**Published:** 1999-03

**Authors:** S Majumdar, E P Diamandis

**Affiliations:** Department of Pathology and Laboratory Medicine, Mount Sinai Hospital, Toronto, Ontario, M5G 1L5, Canada

**Keywords:** prostate specific antigen gene, mutations, promoter, enhancer, breast cancer

## Abstract

KLK3 or prostate specific antigen (PSA) is a serine protease, which is an established tumour marker of prostatic adenocarcinoma. PSA is now used widely for the diagnosis and monitoring of patients with prostate cancer. Recent studies have demonstrated that about 70% of breast cancers produce PSA. In this study, we examined the molecular mechanism underlying the expression of the PSA gene in breast cancer and breast cancer cell lines. We analysed nine breast tumours categorized on the basis of high- or low-PSA expression in tumour cytosols and four breast cancer cell lines. To determine abnormalities associated with PSA expression in breast tumours, genomic DNA was extracted and all five exons of the PSA gene were polymerase chain reaction (PCR) amplified and sequenced on both strands. PCR amplification was also performed for the promoter and enhancer elements of the PSA gene. No mutations were observed in the coding portion of the gene. A polymorphism was observed in exon 2 from three breast tumours. However, sequencing of the promoter and the enhancer elements of the PSA gene reveals several point mutations. Within a 5.8-kb promoter/enhancer region of the PSA gene, we detected 16 different mutational hotspots (appearing more than once in the nine tumours). Among these hotspots, two appeared in seven out of nine tumours. Most importantly, the androgen response element (ARE I) in the proximal promoter was found mutated in four tumours and in the breast carcinoma cell line MCF-7. Mutations associated with the ARE I have been shown previously to result in an 80% decrease in PSA gene expression. The mutations in the core enhancer and promoter region probably contribute to the aberrant expression of the PSA gene in breast tumours, possibly by altering the regulation of the gene by steroid hormones. © 1999 Cancer Research Campaign


*British Journal of Cancer* (1999) **79**(9/10), 1594–1602
© 1999 Cancer Research Campaign
Article no. bjoc.1998.0254

### The promoter and the enhancer region of the KLK 3 (prostate specific antigen) gene is frequently mutated in breast tumours and in breast carcinoma cell lines

**S Majumdar and EP Diamandis**

Department of Pathology and Laboratory Medicine, Mount Sinai Hospital, Toronto, Ontario M5G 1X5, Canada; Department of Laboratory Medicine and Pathobiology, University of Toronto, Toronto, Ontario M5G 1L5, Canada

**Summary** KLK3 or prostate specific antigen (PSA) is a serine protease, which is an established tumour marker of prostatic adenocarcinoma. PSA is now used widely for the diagnosis and monitoring of patients with prostate cancer. Recent studies have demonstrated that about 70% of breast cancers produce PSA. In this study, we examined the molecular mechanism underlying the expression of the PSA gene in breast cancer and breast cancer cell lines. We analysed nine breast tumours categorized on the basis of high- or low-PSA expression in tumour cytosols and four breast cancer cell lines. To determine abnormalities associated with PSA expression in breast tumours, genomic DNA was extracted and all five exons of the PSA gene were polymerase chain reaction (PCR) amplified and sequenced on both strands. PCR amplification was also performed for the promoter and enhancer elements of the PSA gene. No mutations were observed in the coding portion of the gene. A polymorphism was observed in exon 2 from three breast tumours. However, sequencing of the promoter and the enhancer elements of the PSA gene reveals several point mutations. Within a 5.8-kb promoter/enhancer region of the PSA gene, we detected 16 different mutational hotspots (appearing more than once in the nine tumours). Among these hotspots, two appeared in seven out of nine tumours. Most importantly, the androgen response element (ARE I) in the proximal promoter was found mutated in four tumours and in the breast carcinoma cell line MCF-7. Mutations associated with the ARE I have been shown previously to result in an 80% decrease in PSA gene expression. The mutations in the core enhancer and promoter region probably contribute to the aberrant expression of the PSA gene in breast tumours, possibly by altering the regulation of the gene by steroid hormones.

**Keywords:** prostate specific antigen gene; mutations; promoter; enhancer; breast cancer

One of the most serious health problems affecting women is breast cancer. The incidence of breast cancer has risen at an alarming rate over the past 40 years. Breast cancer-associated deaths will rise to 500 000 every year by the year 2000 (Pisani et al, 1985). Recent studies have shown that inherited or sporadic mutation in the breast cancer susceptibility genes *BRCA1* and *BRCA2* account for 45% of increased incidence of the disease. The mechanisms associated with the loss of function of these genes and its correlation with disease incidence is not exactly known, due primarily to the absence of knowledge pertaining to their function. Therefore, the identification and use of novel approaches for the prevention and detection of breast cancer are urgently needed.

KLK3, commonly known as prostate specific antigen (PSA), is an established marker for prostate cancer (Stamey et al, 1987; Catalona et al, 1991). It was originally identified as a tissue-specific protein expressed exclusively by the epithelial cells of the prostate gland (Oesterling, 1991). Recently, improved ultrasensitive methods and RNA analysis has shown that KLK3 (PSA) is not exclusively synthesized by the human prostate gland, but also found to be produced by the breast, ovary, liver, kidney, adrenal and parotid glands (Levesque et al, 1995; Smith et al, 1995). With ultrasensitive immunofluorometric PSA assays, which have a detection limit of 1 ng $l^{-1}$, it has been observed that > 50% of female sera have detectable PSA concentration (Ferguson et al, 1996). PSA can be measured in ~70% of breast cancer cytosolic extracts (Ferguson et al, 1996). A number of human breast cancer cell lines have also been described to express this glycoprotein in a hormone-dependent manner (Yu et al, 1994; Zarghami et al, 1997).

The human kallikrein gene family consists of three members: prostate specific antigen (PSA; KLK3), glandular kallikrein (GK-1; KLK2) and pancreatic/renal kallikrein (KLK1) (Schedlich et al, 1987; MacDonald et al, 1988; Digby et al, 1989; Riegman et al, 1989). These three genes are clustered in an area of 60 kb on human chromosome 19q13.2–13.4 (Riegman et al, 1992). PSA, a kallikrein-like protease, was originally found to be synthesized by normal, hyperplastic and malignant prostatic epithelium, and is thought to be required for the liquefaction of seminal clot after ejaculation (Lilja, 1985; Watt et al, 1986). PSA exists primarily in two immunoreactive forms: a ~ 30-kDa monomer (free PSA) and a ~100-kDa complex with the protease inhibitor $\alpha_1$-anti-chymotrypsin (PSA-ACT) (McCormak et al, 1995).

The PSA gene consists of five functionally coded exons with an open reading frame of 257 amino acids encoding a protein with a predicted molecular weight of ~30 kDa. The promoter and the enhancer elements of the PSA gene have been extensively characterized (Schuur et al, 1996; Cleutjens et al, 1997). The 5′ flanking

*Received 16 February 1998*
*Accepted 13 July 1998*

*Correspondence to:* EP Diamandis, Department of Pathology and Laboratory Medicine, Mount Sinai Hospital, 600 University Avenue, Toronto, Ontario M5G 1X5, Canada

 © Cancer Research Campaign 1999

portion of the gene is approximately 6 kb long and located 12 kb upstream of the hGK-1 gene. The PSA gene is under regulation by steroid hormones. Studies have shown that under physiological and pathological situations, PSA production is a steroid hormone receptor-mediated response. Others have shown that the wild-type PSA gene is regulated by the androgen receptor (AR), mediating transcription by binding to its cognate sequence on the PSA proximal promoter at position –154 and –394 (Schuur et al, 1996). In addition, a third androgen response element (ARE) is located in the distal enhancer at position –4200 (Cleutjens et al, 1997). The proximal and the distal enhancer of the PSA gene interact in a cooperative manner and are required for optimal expression of the gene (Riegman et al, 1991; Schuur et al, 1996; Cleutjens et al, 1997). The ARE-I and -III elements are high-affinity binding sites accounting for 80% of promoter activity (Schuur et al, 1996; Cleutjens et al, 1997).

Steroid hormone receptors are known to recognize common hormone response elements (HREs). DNA binding analysis such as DNAse protection and methylation interference studies of the MMTV promoter shows nearly identical binding patterns for AR and glucocorticoid receptor (GR) (Ahe et al, 1985; Truss and Beato, 1993; Cairns et al, 1991). Similar studies with the tyrosine aminotransferase (TAT) gene shows AR, GR and progesterone receptor (PR) binding to the same HRE sequence in the gene. Studies from our laboratory have shown that the breast carcinoma cell lines T-47D and BT-474, upon induction with androgens and progestins, produce large amounts of PSA (Zarghami et al, 1997). Therefore, it would appear that the PR receptor mediates its effect through the androgen response elements in the PSA gene.

Because the PSA gene is an important clinical marker and its widespread expression in various organs is under regulation by steroid hormones, expression and regulation of the PSA gene in breast cancer might signify the involvement of the gene during progression, pathogenesis or under certain stages of the disease. In fact, there are already indications that PSA is a marker of prognosis and risk assessment in breast cancer patients (Yu et al, 1995; Sauter et al, 1996). In an earlier study by Tsuyuki et al (1997), 1.4 kb of the PSA promoter region displayed multiple base substitutions and deletions with no mutations in the cDNA of the gene. Because the enhancer element of the PSA gene appears to be critical for gene expression, we were interested to characterize the coding and promoter/enhancer region of the PSA gene from a subset of breast tumours and also breast carcinoma cell lines for which the PSA and PR and oestrogen receptor (ER) status was known. This would allow us to systematically determine any mutations that are frequently observed in the gene, especially in the HREs, and to correlate them with the presence or absence of PSA.

**Table 1** Concentration of PSA in breast tumour extracts

| Tumour no. | ER (fmol mg$^{-1}$) | PR (fmol mg$^{-1}$) | PSA (ng g$^{-1}$) |
|---|---|---|---|
| 1 | 0 | 0 | 5248 |
| 2 | 531 | 674 | 0 |
| 3 | 3 | 5 | 7141 |
| 4 | 112 | 0 | 9291 |
| 5 | 634 | 0 | 348 |
| 6 | 98 | 1230 | 0 |
| 7 | 389 | 436 | 0 |
| 8 | 410 | 374 | 0 |
| 9 | 32 | 689 | 0 |

### MATERIALS AND METHODS

### Breast tumours

Approximately 1500 breast tumour cytosolic extracts were screened for PSA protein levels using a highly sensitive immunofluorometric assay (Ferguson et al, 1996). These tumours were also analysed for oestrogen receptor (ER) and progesterone receptor (PR) protein levels as part of routine investigations. Out of the 1500 tumours, nine tumours were selected for this study that displayed variable amounts of PSA protein ranging from 0 ng g$^{-1}$ to 9291 ng g$^{-1}$ with corresponding low and high levels of oestrogen and progesterone receptor (Table 1).

### Cell lines

We examined four breast carcinoma cell lines which were cultured as described previously (Zarghami et al, 1997). The receptor status and inducibility of PSA production by steroid hormones in these cell lines is shown in Table 2.

### Genomic DNA extraction

Genomic DNA from tumours was extracted according to the standard proteinase K digestion and phenol/chloroform extraction method (Maniatis et al, 1982). Briefly, the tumour samples were pulverized with a prechilled mortar and pestle to a fine powder. The powdered tissue was digested with digestion buffer containing 100 mM sodium chloride, 10 mM tris-HCl, pH 8, 25 mM EDTA, pH 8, 0.5% sodium dodecyl sulphate, 0.1 mg ml$^{-1}$ proteinase K and incubated at 50°C with shaking for 12–18 h. For extraction of nucleic acids, the digested samples were extracted with an equal volume of phenol/chloroform/isoamyl alcohol and centrifuged for

**Table 2** Breast carcinoma cell lines used in this study and their ability to produce PSA

| Cell line | ER status | PR status | PSA production |
|---|---|---|---|
| T-47D | + | + | Relatively large amounts of PSA produced after stimulation with androgens, progestins, glucocorticoids and mineralocorticoids, but not oestrogens |
| MCF-7 | + | + | Traces of PSA produced after stimulation by glucocorticoids and progestins, but not androgens or oestrogens |
| BT-474 | + | + | Relatively large amounts of PSA produced after stimulation by androgens, progestins, mineralocorticoids and smaller amounts by glucocorticoids and oestrogens |
| ZR-75-1 | + | + | No PSA production after stimulation with any steroid hormones |

© Cancer Research Campaign 1999

10 min at 1700 ***g***. The aqueous phase was then transferred to a new tube and the DNA precipitated with 0.5 volumes of 7.5 M ammonium acetate and two volumes of ethanol.

Genomic DNA isolated from lymphocytes was extracted as follows: red blood cells were lysed with 30 ml of lysis buffer (155 mM ammonium chloride, 10 mM potassium bicarbonate, 1 mM disodium EDTA, pH 8.2), incubated on ice for 15 min and centrifuged at 2000 r.p.m. for 10 min. The supernatant was discarded and 10 ml of lysis buffer was added and centrifugation repeated as above. Three millilitres of nuclei lysis buffer was added and digested overnight at 37°C. Three millilitres of sterile water and 3 ml of 6 M sodium chloride was added and mixed vigorously to precipitate proteins and spun at 13 000 r.p.m. for 3 min. The DNA was precipitated by adding two volumes of 100% ethanol. The DNA was spooled out and transferred to a microfuge tube and dissolved in TE buffer (10 mM tris-HCl, 0.2 mM disodium EDTA, pH 7.5).

### Polymerase chain reaction (PCR) amplification

PCR amplification of the PSA exons were performed as follows: one cycle of denaturation at 94°C for 5 min followed by denaturation at 94°C for 30 s, annealing at 65°C for 30 s and extension at 72°C for 30 s, repeated for 30 cycles. The final cycle was an extension for 5 min at 72°C. The primer sequences used for the amplification of the individual exons are listed in Table 3. Sequencing primers for these fragments are shown in Table 4.

PCR amplification of the PSA promoter and enhancer elements was carried out with nine specific pairs of primers as shown in Table 5, and sequencing primers are presented in Table 6. Fragment numbers 1, 2, 3, 5 and 6 were amplified essentially as described above. Fragment numbers 4, 7 and 9 were similarly amplified with an annealing temperature of 60°C.

### Sequencing

The five exons and the promoter and enhancer elements of the PSA gene were PCR amplified from the various breast tumours

**Table 3** PCR primers for the amplification of the PSA exons

| Exon | Primer name | Sequence[a] | PCR fragment size (basepairs) | Coordinates[b] |
|---|---|---|---|---|
| 1 | 1 | ATGGGGAGGGCCTTGGTCAG | 281 | 539 to 558 |
| | 1′ | TGTCTGGGCTGGGGTGCTG | | 801 to 819 |
| 2 | 2 | CCCCCGTGTCTTTTCAAACC | 407 | 1856 to 1875 |
| | 2′ | GCCTCCCCATGTGACCTGA | | 2244 to 2262 |
| 3 | 3 | CCCAACCCTGTGTTTTTCTC | 501 | 3634 to 3653 |
| | 3′ | GGCCCTCCTCCCTCAGA | | 4118 to 4134 |
| 4 | 4 | GGAGGAGGGGACAGGACTC | 302 | 4094 to 4112 |
| | 4′ | TCTAGACCCCAGCCCAGAAT | | 4376 to 4395 |
| 5 | 5 | GTCGGCTCTGGAGACATTTC | 374 | 5558 to 5577 |
| | 5′ | AACTGGGGAGGCTTGAGTC | | 5613 to 5631 |

[a]All sequences are given in the 5′–3′ direction. [b]Refers to nucleotide numbers based on Genbank accession no. M27274.

**Table 4** Primers used for the sequencing of the PSA exons

| Exon | Primer name | Sequence[a] | Coordinates[b] |
|---|---|---|---|
| 1 | 1s | GAGTCCTGGGGAATGAA | 586 to 602 |
| | 1as | GAAAGAGCCTCAGCTTG | 774 to 790 |
| 2 | 2s | TCCATCTCCTATCCGAG | 1887 to 1903 |
| | 2as | CAGAACTTTCCCTCTCT | 2225 to 2241 |
| 3 | 3s | TCTCTTTTGGAGCCTCC | 3666 to 3682 |
| | 3as | AGGAGTCCTGTCCCCTC | 4098 to 4114 |
| 4 | 4s | TGAGGGAGGAGGCCCAA | 4120 to 4136 |
| | 4as | TTAAGGTCCCCACTCAC | 4360 to 4376 |
| 5 | 5s | TCCAAAGCTGGGAACTG | 5586 to 5602 |
| | 5as | GGCCTGGTCATTTCCAA | 5894 to 5910 |

[a]All sequences are given in the 5′–3′ direction. [b]Refers to the nucleotide numbers based on Genbank accession no. M27274. s, sense; as, antisense.

 © Cancer Research Campaign 1999

**Table 5** Primer sequences used for the PCR amplification of the PSA promoter and enhancer elements

| Primer name | Sequence[a] | Coordinates[b] |
|---|---|---|
| 1 | TTTCCCGGTGACATCGTG | – 5768 to – 5751 |
| 1′ | CTCAAAGACCTCAGGCGTTTC | – 5001 to – 5021 |
| 2 | AAGAGATGACCTCTCAGGCTC | – 5086 to – 5066 |
| 2′ | TGACTCAGGAGTCTCATGGAC | – 4394 to – 4414 |
| 3 | TTTGTGAATGCTGGCAGAG | – 4432 to – 4413 |
| 3′ | CCTTTGAAATTCAAGAGGGTG | – 3650 to – 3670 |
| 4 | GGTCCCGATCGACTGTGTCTG | – 3790 to – 3770 |
| 4′ | GCAGGCCACTCCCACCAG | – 3082 to – 3099 |
| 5 | CAGCCACCTGCCAACCGTAGA | – 3135 to – 3115 |
| 5′ | TCTGAGCCAACATCAAAACAA | – 2398 to – 2418 |
| 6 | AAATTAGCTGGGCATGGTGCT | – 2453 to – 2435 |
| 6′ | GTTTGGGATGGCATGGCTT | – 1765 to – 1785 |
| 7 | TAGAAACAGGGGAAGATTTTA | – 1947 to – 1967 |
| 7′ | ATTACAGGTGCCCTGTG | – 1078 to – 1094 |
| 8 | CCGGGCGTGGTGGCAC | – 1092 to – 1107 |
| 8′ | TCCCTGATCCCTGCACCACTC | – 384 to – 404 |
| 9 | TTTCAGGAGCATGAGGAATAA | – 426 to – 446 |
| 9′ | CCCAGGAGCCCTATAAAAC | – 12 to – 30 |

[a]All sequences are given in the 5′–3′ direction. [b]Refers to the nucleotide numbers based on Genbank accession no. U37672.

**Table 6** Primer sequences used for the sequencing of the PSA promoter and enhancer elements

| Primer name | Sequence[a] (X = Cy5 label) | Coordinates[b] |
|---|---|---|
| 1 | X-TGACATCGTGGAAAGCAC | – 5760 |
| 2 | X-TCAGGAGACTGAGACCAT | – 5448 |
| 3 | X-TTAGCCAGGATGGTCTCA | – 5457 |
| 4 | X-TCAGGCGTTTCCCCTCAG | – 5066 |
| 5 | X-TGACCTCTCAGGCTCTGA | – 5080 |
| 6 | X-CTGCAGTTGGTGAGTGGT | – 4730 |
| 7 | X-AGAGACCATGACCACTAC | – 4740 |
| 8 | X-TCTCATGGACTCTGCCAG | – 4422 |
| 9 | X-TGCTGGCAGAGTCCATGA | – 4424 |
| 10 | X-CTTGCAAGATGATATCT | – 4030 |
| 11 | X-TCTGAGAGAGATATCATC | – 4038 |
| 12 | X-TTCAAGAGGGTGGACCCA | – 3676 |
| 13 | X-ACTGTGTCTGGCAGCACT | – 3779 |
| 14 | X-ACCTAATGGTTCCAGCCT | – 3433 |
| 15 | X-AAGGCAGAGGCTGGAACC | – 3440 |
| 16 | X-ACCAGAGGAGGATGGGCA | – 3112 |
| 17 | X-AACCGTAGAGCTGCCCAT | – 3123 |
| 18 | X-TCAATCCTATACCCAGCA | – 2654 |
| 19 | X-TTAGATAAAGTGCTGGGT | – 2660 |
| 20 | X-AAAACAAATCTATTTCCA | – 2429 |
| 21 | X-ATGGCTTTGGGATGGAAA | – 2441 |
| 22 | X-TTCCCTGGATTCATCCTG | – 2086 |
| 23 | X-TTCCTTTCCAGGATGAAT | – 2094 |
| 24 | X-ATGGTGCTGCATGCCTGT | – 1795 |
| 25 | X-AAGATTTTATCAAATTCT | – 1955 |
| 26 | X-TACCTCCACTGTTAACTC | – 1527 |
| 27 | X-TTTGCATGCCACCTTAAT | – 1459 |
| 28 | X-TTCTGGGTTCAAGTGATT | – 1048 |
| 29 | X-TGAACCCAGAAGGCAGA | – 1041 |
| 30 | X-AATAACCCCCCACTCCTT | – 652 |
| 31 | X-AATTCCACATTGTTTGCT | – 632 |
| 32 | X-TAGATCCTTTGCACTCCA | – 337 |
| 33 | X-AAGGATCTAGGCACGTGA | – 328 |
| 34 | X-TGGGGCTGGGGAGCCTCC | – 11 |

[a]All sequences are given in the 5′–3′ direction. [b]Refers to the nucleotide numbers based on Genbank accession no. U37672.

and cell lines as described above and sequenced directly. The PCR reaction products were purified by Qiagen quick PCR purification columns (Qiagen, Clatsworth, CA, USA) to remove excess primers and deoxynucleosides. The details of the different oligonucleotides used for sequencing of the PSA exons and 5′ flanking sequences are outlined in Tables 4 and 6 respectively. The sequencing primers were Cy5 fluorescent dye labelled at the 5′ end. Sequencing on the purified PCR fragments was performed using the Thermosequenase Cycle Sequencing protocol (Amersham Life Sciences, Buckinghamshire, UK), the fragments were then resolved on a 6% sequencing gel on the ALF Express automated Sequencer (Pharmacia Biotech, Sweden). Sequencing was performed on both strands of DNA. Any mutations identified were further verified by additional PCR reactions followed by sequencing.

### RESULTS

### Expression of the PSA gene in tumours with undetectable PSA protein

The breast tumours selected for this study showed variable levels of PSA expression associated with presence or absence of ER and PR. To determine whether the tumours with 0 ng $g^{-1}$ PSA harboured any mutations in the coding region of the PSA gene, the five PSA exons were PCR amplified with specific primers (Table 3), and the PCR reactions were run on agarose gels. All the PSA exons from all nine tumours and cell lines amplified efficiently and to their correct sizes as determined by agarose gel electrophoresis (Figure 1). The PCR amplified reactions were then directly sequenced with Cy5-labelled primers (Table 4) to determine any mutations. No abnormalities were observed in the protein-coding portion of the gene. The PCR sequences were identical to the corresponding region of the PSA mRNA sequence registered in GenBank (accession no. M27274). In three out of nine tumours, a polymorphism was observed in exon 2 (Figure 2), as also reported previously (Schulz et al, 1988; Lundwall, 1989; Tsuyuki et al, 1997). This suggests that the coding region of the PSA gene is not a target for mutations in breast cancer.

*© Cancer Research Campaign 1999*

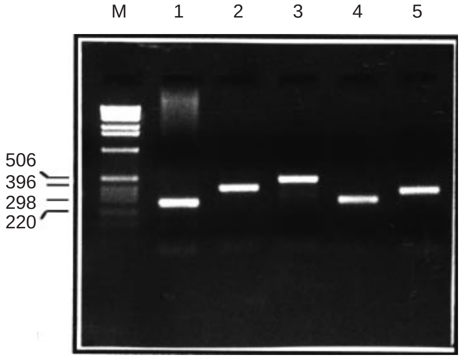

**Figure 1** Agarose gel electrophoresis of PCR-amplified PSA exons 1–5. PCR was performed as described in the Materials and methods section. The PCR reaction products were run on a 2% agarose gel stained with ethidium bromide and viewed under UV light. The numbers on the top of the gel represent the number of the exon. M represents the 1-kb molecular weight standard. The length of the PCR products are 281 bp (exon 1), 407 bp (exon 2), 501 bp (exon 3), 302 bp (exon 4) and 374 bp (exon 5)

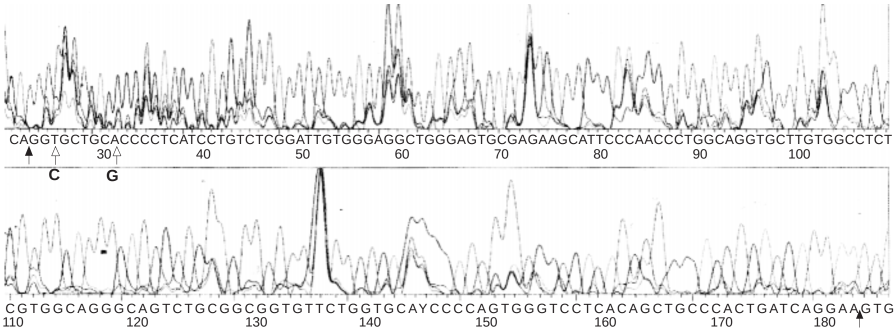

**Figure 2** PSA exons amplified by PCR were sequenced on the ALF Express automated Sequencer using Cy5-labelled primers. A polymorphism was observed in exon 2 in three breast tumours corresponding to the sequence published by Schulz et al (1988). The filled arrows indicate the beginning and end of the exon. The open arrows indicate the sites of the polymorphism. In the wild-type sequence, the nucleotides are T and A, and in the polymorphic sequences are C and G

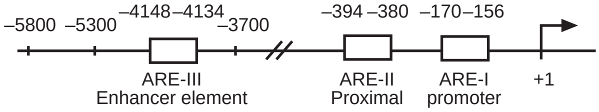

**Figure 3** Schematic representation of the PSA gene promoter and enhancer elements. The position of the AREs (androgen response elements) and the respective coordinates are indicated. The enhancer element is located between positions – 3.7 kb and – 5.3 kb. The transcription start site is indicated as +1. The figure is not drawn to scale

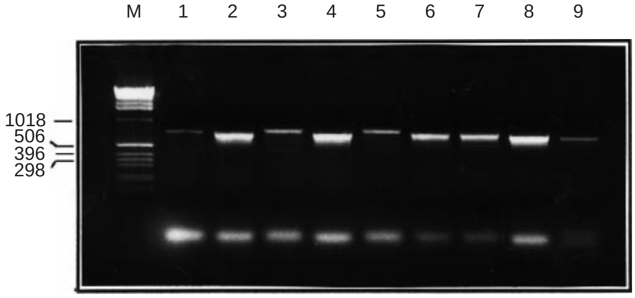

**Figure 4** PCR amplification of the promoter and enhancer elements of the PSA gene. The PCR products were run on a 2% agarose gel stained with ethidium bromide. The 5.8-kb PSA 5′-flanking region was amplified using nine pairs of PCR primers. Each fragment amplified was approximately 700–800 bp in length. The numbers above each lane represent the primer pair used to amplify that fragment (details of primers and coordinates are given in Table 4). M represents the 1-kb molecular weight marker

### Mutations in the upstream regulatory element of the PSA gene in breast cancer

To examine whether the variable expression of the PSA protein is determined at the level of regulation, the PSA promoter and enhancer element were PCR amplified using nine different pairs of primers (primers designed from the wild-type gene sequence as published by Schuur et al, 1996) encompassing 5.8 kb of upstream sequences from all the tumours and breast cancer cell lines (Table 5). A schematic representation of the PSA promoter and enhancer with the ARE elements are depicted in Figure 3. The

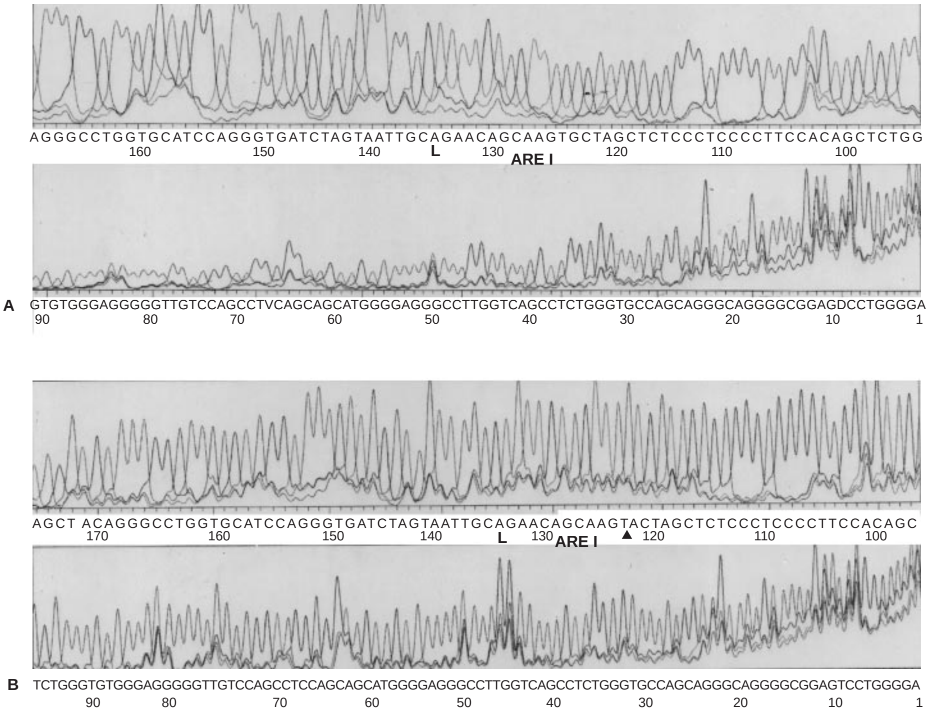

**Figure 5** A representative example of the wild-type ARE-I (**A**) and the mutated ARE-I (**B**) which was observed in four tumours and in the breast cancer cell line MCF-7. A point mutation (G–A) was observed in the half site of the ARE-I element between –156 and –170 in the PSA promoter. The mutation in the lower panel is indicated by a black triangle

PCR products, each approximately 700–800 bases, were analysed on agarose gels (Figure 4). PSA promoter and enhancer characterization was performed by DNA sequence analysis on both strands. Each of the PCR-amplified fragments was directly sequenced with specific Cy5-labelled primers (Table 6) on an automated sequencer. We observed numerous deletions, insertions and point mutations in comparison with the wild-type sequence in all the breast tumours and cell lines. The distinct mutations in the various tumours are shown in detail in Table 7 along with the corresponding PSA, ER and PR protein levels in the tumour cytosols. The mutations observed more than once in the tumours and cell lines are outlined in Table 8. From previous studies by Schuur et al (1996) and Cleutjens et al (1997), it is apparent that the ARE-I is a critical regulatory element of the PSA gene. Interestingly, the ARE-I in a proportion of breast tumours and in the breast carcinoma cell line MCF-7 was observed to be frequently altered in the half site.

### DISCUSSION

The complex molecular mechanisms underlying the development and progression of breast cancer are not very well understood. Studies have uncovered genes that are responsible for familial and sporadic breast cancer. Recently, we identified an important prostatic tumour marker, which is also expressed in normal, hyperplastic and cancerous breast tissue. PSA or KLK3 appears to be a favourable prognostic indicator for breast cancer (Yu et al, 1995). This study is the first to demonstrate multiple mutations in the enhancer element of the PSA gene in breast cancer.

To determine the molecular mechanism by which the PSA gene is regulated in breast cancer, we selected tumours that expressed variable levels of PSA protein and progesterone receptor. To determine whether tumours with undetectable PSA protein were associated with mutations in the PSA gene, we PCR amplified the five PSA exons from genomic DNA extracted from the tumours and analysed the PCR products by sequencing. This sequence analysis revealed the absence of mutations in any of the five PSA exons. These data agree with our previously published observations regarding the mutational status of the PSA coding exons and a small part of the proximal promoter of this gene (Tsuyuki et al, 1997). There are several other possible explanations for the aberrant PSA expression in the breast tumours and cell lines that we analysed. An upstream regulator involved in the negative regulation of the gene might result in low or undetected expression of the PSA gene. A second possibility is that the PSA gene becomes inactivated as the tumour either dedifferentiates or undergoes an abnormal developmental programme. These explanations are not mutually exclusive because changes in control of expression of the PSA gene by upstream factors may result from an altered developmental programme.

Another important possibility is that an epigenetic change such as methylation could have inactivated the locus. In breast cancer, methylation of 5′ sequences in other genes is associated with loss of activity of these genes (Ottaviano et al, 1994; Graff et al, 1995; Yoshiura et al, 1995). Methylation can also result in another type

*© Cancer Research Campaign 1999* 

**Table 7** Novel mutations in the PSA gene promoter/enhancer elements in breast tumours and cell lines, found only once

| Tumour/cell line | ER | PR | PSA | Mutations identified[a] | |
|---|---|---|---|---|---|
| 1 | 0 | 0 | 5248 | G–A[b] | – 3900 |
| | | | | G–T | – 2590 |
| | | | | Insertion of G | – 2821 |
| 2 | 531 | 674 | 0 | C–T | – 4759 |
| | | | | A–C | – 4279 |
| | | | | T–A | – 4002 |
| | | | | Insertion of T | – 2919 |
| | | | | Insertion of G | – 2916 |
| | | | | Insertion of G | – 2069 |
| | | | | Insertion of CAA | – 1908 |
| 4 | 112 | 0 | 9291 | Deletion of G | – 4774 |
| | | | | Insertion of C | – 4768 |
| | | | | G–A | – 4606 |
| | | | | Insertion of C | – 352 |
| | | | | Deletion of A | – 155 |
| 5 | 634 | 0 | 348 | A–C | – 4219 |
| | | | | Insertion of T | – 4066 |
| | | | | A–G | – 3927 |
| | | | | Deletion of G | – 2814 |
| | | | | Deletion of C | – 2485 |
| | | | | A–G | – 155 |
| 6 | 98 | 1230 | 0 | Insertion of C | – 3882 |
| | | | | G–C | – 3798 |
| | | | | A–C | – 3147 |
| | | | | Insertion of C | – 2810 |
| | | | | C–T | – 2619 |
| | | | | Insertion of G | – 2457 |
| | | | | Insertion of G | – 1762 |
| | | | | Deletion of C | – 1369 |
| | | | | Insertion of C | – 692 |
| | | | | T–G | – 511 |
| 7 | 389 | 436 | 0 | Insertion of G | – 5581 |
| | | | | Insertion of T | – 5537 |
| | | | | Insertion of G | – 5117 |
| | | | | T–G | – 4579 |
| | | | | A–C | – 4272 |
| | | | | Insertion of C | – 2968 |
| | | | | Deletion of A | – 2599 |
| 8 | 410 | 374 | 0 | G–A | – 5261 |
| | | | | A–C | – 4976 |
| | | | | C–T | – 4866 |
| | | | | Insertion of G | – 2939 |
| | | | | Deletion of C | – 2241 |
| | | | | T–G | – 1212 |
| MCF-7 | N/A[c] | N/A | N/A | Insertion of T | – 5488 |
| | | | | Insertion of G | – 5449 |
| | | | | A–G | – 4142 |
| | | | | G–T | – 4041 |
| | | | | A–G | – 252 |
| | | | | Deletion of A | – 206 |
| T-47D | N/A | N/A | N/A | Insertion of T | – 5503 |
| | | | | Insertion of G | – 319 |
| BT-474 | N/A | N/A | N/A | Insertion of T | – 4038 |
| | | | | A–G | – 3316 |
| | | | | Insertion of T | – 3313 |
| | | | | Insertion of G | – 3278 |
| | | | | Insertion of C | – 3203 |
| | | | | Deletion of GTG | – 3190 |
| ZR-75-1 | N/A | N/A | N/A | C–G | – 5600 |
| | | | | T–C | – 5428 |
| | | | | Deletion of A | – 5169 |
| | | | | A–C | – 4733 |
| | | | | Deletion of G | – 3611 |
| | | | | C–G | – 2942 |

[a]Refers to nucleotide numbers in Genbank accession no. U37672. [b]Indicates that the mutation is in the core enhancer. [c]N/A, not applicable; see Table 2.

 *© Cancer Research Campaign 1999*

**Table 8** Mutations commonly observed in breast tumours and cell lines

| Coordinates[a] | Mutations | Tumours/cell lines |
|---|---|---|
| – 5703 | G–T | 1, 3 |
| – 5700 | A–T | 1, 2, 4, 5, 6, 7, 8, 9, MCF-7 |
| – 5619 | Insertion of C | 1, 2, 4, 5, 6, 8, 9, MCF-7, T-47D |
| – 5444 | Insertion of T | 1, 3 |
| – 5389 | G–T | 4, 5, 6 |
| – 5378 | Insertion of CAACT | 4, 5, 7 |
| – 4848 | Deletion of T (mutation in core enhancer) | 1, 2 |
| – 4845 | Insertion of G | 1, 2, 3 |
| – 4271 | G–C | 4, MCF-7 |
| – 4269 | A–C | 4, MCF-7 |
| – 3900 | G – A (mutation in core enhancer) | 1, 3 |
| – 3898 | G–A | 5, 6 |
| – 3813 | C–A | 5, 7 |
| – 2669 to – 2745 | Deletion of 76 bases | 1, 2, 3 |
| – 1905 | Deletion of A | 2, 3 |
| – 158 | G–A (ARE-I mutation) | 4, 6, 8, 9, MCF-7 |

[a]Refers to nucleotide numbers based on Genbank accession no. U37672.

of epigenetic event, one that results in regional alteration in chromatin structure other than just primary gene silencing. There is evidence, for example, of patients that show oestrogen receptor negativity due to methylation of the ER receptor (Ottaviano et al, 1994).

To examine the possibility that the PSA gene in breast cancer is under regulation by upstream sequences, we analysed by sequencing the promoter and the enhancer elements of the PSA gene. On PCR amplification of the entire 5.8 kb of regulatory sequences, we identified multiple mutations in the promoter and the enhancer elements. These mutations are presented in Tables 7 and 8. In Table 7, we show mutations that occur only once. The alterations include point mutations, small insertions and small deletions. The single appearance of these genomic changes in tumours and cell lines precludes us to correlate their presence with the expression pattern of PSA, but one striking feature is the frequency of their appearance. Each tumour or cell line has, on average, about six unique nucleotide changes in this region. We speculate that this portion of the genome appears to be a target for multiple genetic changes, contrary to our observation that the coding region of this gene is devoid of such changes. Further functional studies will be necessary to establish whether these and the other changes observed indeed affect the expression of the PSA gene and in which direction.

In Table 8, we report mutations that occurred more than once in either the tumours or the cell lines. Regarding the association of these mutations with PSA protein expression, we have the following comments. The mutation at position –5703 occurs in two tumours that are both progesterone receptor negative and they overexpress PSA. This change may be associated with aberrant PSA gene overexpression that does not require progesterone receptors. The mutation at position –5700 is among the most prevalent, found in eight out of nine tumours examined. This mutation occurs in both steroid hormone receptor (SHR)-positive and -negative tumours and also in PSA(+) and PSA(–) tumours. Strikingly, the appearance of this mutation is always associated with the insertion of C at position –5619 with the exception of tumour 7 and the cell line T-47D, suggesting that these two genetic changes may be linked. The mutations in position –5444 (insertion of T) as well as the mutation at –3900 are seen, similarly to the mutation at –5703, in the two tumours that are receptor negative and at the same time overexpress PSA (tumour numbers 1 and 3). Of possible importance is that one of these mutations (–3900) occurs within the recently identified core enhancer of the PSA gene (Schuur et al, 1996; Cleutjens et al, 1997). The importance of these three mutations (–5703, –5444, –3900) in the aberrant expression of the PSA gene in tumours that have no progesterone receptors needs further investigation.

Among the other mutations, we found no consistency between their presence and PSA expression pattern. For example, the mutations at position –5389, –5378, –4848, –4845, –3898, –3813, –2669 and –1905 are associated with tumours that either overexpress or do not express. Similarly, the mutations at positions –4271 and –4269 appeared only in one tumour and one cell line and could not be correlated with PSA expression.

Among all mutations identified, the one at position –158, which affects the ARE-I, appears to be the most important. It has been previously shown (Henntu et al, 1992; Young et al, 1992) that PSA expression is responsive to androgen stimulation. The regulatory mechanism includes binding of the androgen-activated androgen receptor to the PSA promoter, which contains ARE-I. The ARE-I and ARE-III elements are high-affinity binding sites responsible for > 80% of the promoter/enhancer activity, and a mutational change in ARE-I will probably affect promoter activity and PSA expression as shown previously (Schuur et al, 1996; Cleutjens et al, 1997). The ARE-I mutation was found in four out of nine tumours indicating that it is prevalent. Three of these tumours are steroid hormone receptor positive and yet produce no PSA at all. Also, the MCF-7 cell line, which is steroid hormone receptor positive and does not produce PSA upon androgen stimulation, harbours this mutation as well. We speculate that the presence of this mutation may be associated with loss of androgen-regulated PSA expression in breast tumours.

One tumour (tumour 4), which harbours the ARE-I mutation and is PR(–), produced more PSA than any other tumour in our series. A careful evaluation of the mutational status of this tumour,

© Cancer Research Campaign 1999

and of tumour 5 which has a similar receptor status and also over-expresses PSA, reveals that they both harbour a mutation at nucleotide –155, which lies just outside the consensus sequence of ARE-I (–170 to –156). In the case of tumour 4, the nucleotide at position –155 (A) is deleted and is followed by G. In the case of tumour 5, the mutation changes an A to G. These observations, suggesting aberrant PSA expression associated with genetic changes inside or just outside the ARE-I of the PSA gene promoter, need further investigation with functional studies.

In conclusion, we have presented evidence that the coding region of the PSA gene is not mutated in either breast tumours or breast carcinoma cell lines. We also show that the promoter/enhancer region of this gene, encompassing approximately 5.8 kb of upstream sequence, is a target for numerous mutations, including 16 different mutational hotspots (appearing more than once). Among these hotspots, two appeared in eight out of nine or seven out of nine tumours and one hotspot, within the ARE-I, was found in four out of nine tumours. We are currently pursuing the significance of these mutations and their effect on the expression of the PSA gene by site-directed mutagenesis, followed by functional analysis in transfection studies. We are also examining in a large series of tumours and normal tissues the prevalence of the ARE-I mutation and its association with breast cancer prognosis or susceptibility.

### ACKNOWLEDGEMENTS

We thank Dr J Foekens of the Dr Daniel den Hoed Cancer Centre, The Netherlands, for providing some of the breast tumours utilized in this study.

### REFERENCES

Ahe D, Janich S, Scheidereit C, Renkawitz R, Schutz G and Beato M (1985) Glucocorticoid and progesterone receptors bind to the same sites in two hormonally regulated promoters. *Nature* **313**: 706–709

Cairns C, Gustafsson J-A and Carlstedt-Duke J (1991) Identification of protein contact sites within the glucocorticoid/progestin response element. *Mol Endocrinol* **5**: 598–604

Catalona WJ, Smith DS, Ratliff TL, Dodds KM, Caplen DE, Yuan JJ, Petros JA and Andriole GL (1991) Measurement of prostate-specific antigen in serum as a screening test for prostate cancer. *N Engl J Med* **324**: 1156–1161

Cleutjens KBJM, van der Korput HAGM, van Eekelen CCEM, van Rooij HCJ, Faber PW and Trapman J (1997) An androgen response element in a far upstream enhancer region is essential for high, androgen-regulated activity of the prostate-specific antigen promoter. *Mol Endocrinol* **11**: 148–161

Digby M, Zhang X-Y and Richards RI (1989) Human prostate specific antigen (PSA) gene: structure and linkage to the kallikrein-like gene, hGK-1. *Nucleic Acids Res* **17**: 2137

Ferguson RA, Yu H, Kalyvas M, Zammit S and Diamandis EP (1996) Ultrasensitive detection of prostate specific antigen by time-resolved immunofluorometric assay and the immulite immunochemiluminescent third generation assay: potential applications in prostate and breast cancers. *Clin Chem* **42**: 536–544

Graff JR, Herman JG, Lapidus RG, Chopra H, Xu R, Jarrad DF, Isaacs WB, Pitha PM, Davidson NE and Baylin SB (1995) E-cadherin expression is silenced by DNA hypermethylation in human breast and prostate carcinomas. *Cancer Res* **55**: 5195–5199

Henntu P, Liao S and Vikho P (1992) Androgens upregulate the human prostate specific antigen messenger ribonucleic acid, but down-regulate the prostatic phosphatase mRNA in LNCaP cells. *Endocrinology* **130**: 766–772

Levesque M, Yu H, D'Costa M and Diamandis EP (1995) Prostate specific antigen expression by various tumors. *J Clin Lab Anal* **9**: 123–128

Lilja H (1985) A kallikrein like serine protease in prostatic fluid cleaves the predominant seminal vesicle protein. *J Clin Invest* **76**: 1899–1903

Lundwall A (1989) Characterization of the gene for prostate specific antigen, a human glandular kallikrein. *Biochem Biophys Res Commun* **161**: 1151–1159

McCormak RT, Rittenhouse HG, Finlay JA, Sokoloff, RL, Wang TJ, Wolfert RL, Lilja H and Oesterling JE (1995) Molecular forms of prostate-specific antigen and the human kallikrein gene family: a new era. *Urology* **45**: 729–744

MacDonald RJ, Margolius HS and Erdos EG (1988) Molecular biology of tissue kallikrein. *Biochem J* **253**: 313–321

Maniatis T, Fritsch EF and Sambrook J (1982) *Molecular Cloning: A Laboratory Manual*. Cold Spring Harbor Laboratory Press: Cold Spring Harbor, NY

Oesterling JE (1991) Prostate specific antigen: a critical assessment of the most useful tumor marker for adenocarcinoma of the prostate. *J Urol* **145**: 907–923

Ottaviano YL, Issa JP, Parl FF, Smith HS, Baylin SB and Davidson NE (1994) Methylation of the estrogen receptor gene CpG island marks loss of estrogen receptor expression in human breast cancer cells. *Cancer Res* **54**: 2552–2555

Pisani P, Parkin DM and Ferlay J (1985) Estimates of the worldwide mortality from eighteen major cancers in 1985: implications for prevention and projections of future burden. *Int J Cancer* **55**: 891–903

Riegman PHJ, Vlietstra RJ, van der Korput JAGM, Romijn JC and Trapman J (1989) Characterization of the prostate specific antigen gene: a novel human kallikrein-like gene. *Biochem Biophys Res Commun* **159**: 95–102

Riegman PHJ, Vliestra RJ, Korput JAGM, Romijn JC and Trapman J (1991) Identification and androgen regulated expression of two major human glandular kallikrein-1 (hGK-1) mRNA species. *Mol Cell Endocrinol* **76**: 181–190

Riegman PHJ, Vlietstra RJ, Suurmeijer L, Cleutjens CBJM and Trapman J (1992) Characterization of the human kallikrein locus. *Genomics* **14**: 6–11

Sauter ER, Daly M, Lenahan K, Ehya H, Engstrom PF, Sorling A, Bonney G, Yu H and Diamandis EP (1996) Prostate-specific antigen levels in nipple aspirate fluid correlate with breast cancer risk. *Cancer Epidemiol Biomarkers Prev* **5**: 967–970

Schedlich LJ, Bennetts BH and Morris BJ (1987) Primary structure of a human glandular kallikrein gene. *DNA* **6**: 429–437

Schulz P, Stucka R, Feldmann H, Combriato G, Klobeck H-G and Fittler F (1988) Sequence of a cDNA clone encompassing the complete mature human prostate specific antigen (PSA) and an unspliced leader sequence. *Nucleic Acids Res* **16**: 6226

Schuur ER, Henderson GA, Kmetec LA, Miller JD, Lamparski HG and Henderson DR (1996) Prostate-specific antigen expression is regulated by an upstream enhancer. *J Biol Chem* **271**: 7043–7051

Smith MNR, Biggar S and Hussain M (1995) Prostate specific antigen messenger RNA is expressed in non-prostate cells: implications for detection of micrometastases. *Cancer Res* **55**: 2640–2644

Stamey TA, Yang N, Hay AR, McNeal JE, Freiha FS and Redwine E (1987) Prostate specific antigen as a serum marker for adenocarcinoma of the prostate. *N Engl J Med* **317**: 909–916

Truss M and Beato M (1993) Steroid hormone receptors: interaction with deoxyribonucleic acid and transcription factors. *Endocrinol Rev* **14**: 459–479

Tsuyuki D, Grass L, Diamandis EP (1997) Frequent detection of mutations in the 5′ flanking region of the prostate specific antigen gene in female breast cancer. *Eur J Cancer* **33**: 1853–1856

Watt KW, Lee PJL, M'Timkulu T, Chan WP and Loor R (1986) Human prostate-specific antigen: structural and functional similarity with serine protease. *Proc Natl Acad Sci USA* **83**: 3166–3170

Yoshiura K, Kanai Y, Ochiai A, Shimoyama Y, Sugimura T and Hirohashi S (1995) Silencing of the E-cadherin invasion-suppressor gene by CpG methylation in human carcinomas. *Proc Natl Acad Sci USA* **92**: 7416–7419

Young CYF, Andrews PE, Montgomery BT and Tindall DJ (1992) Tissue specific and hormonal regulation of human prostate-specific glandular kallikrein. *Biochemistry* **31**: 818–824

Yu H, Diamandis EP, Zarghami N and Grass L (1994) Induction of prostate specific antigen production by steroid and tamoxifen in breast cancer cell lines. *Breast Cancer Res Treat* **32**: 291–300

Yu H, Diamandis EP, Katsaros D, Sutherland DJA, Levesque MA, Roagna R, Ponzone R and Sismondi P (1995) Prostate-specific antigen is a new favorable prognostic indicator for women with breast cancer. *Cancer Res* **55**: 2104–2110

Yu H, Diamandis EP, Levesque M, Asa SL, Monne M and Croce CM (1995) Expression of the prostate specific gene by a primary ovarian carcinoma. *Cancer Res* **55**: 1603–1606

Zarghami N, Grass L and Diamandis EP (1997) Steroid hormone regulation of prostate specific antigen gene expression in breast cancer. *Br J Cancer* **33**: 579–588

*© Cancer Research Campaign 1999*
